# Cross-Cultural Contact and Norwegian Language Skills Among Ethnic Minority Women in Norway, and Relationship with Physical Activity in Pregnancy and Postpartum: The STORK-Groruddalen Cohort Study

**DOI:** 10.1007/s10903-023-01535-9

**Published:** 2023-08-28

**Authors:** Karin Elisabeth Bennetter, Christin Wiegels Waage, Anne Karen Jenum, Nina Køpke Vøllestad, Hilde Stendal Robinson, Kåre Rønn Richardsen

**Affiliations:** 1 Institute of Health and Society, Department of General Practice, Faculty of Medicine, University of Oslo, Box 1130, 0318 Blindern, Oslo, Norway; 2 Institute of Physiotherapy, Faculty of Health Science, Oslo Metropolitan University, Oslo, Norway; 3 General Practice Research Unit (AFE), Department of General Practice, Institute of Health and Society, Faculty of Medicine, University of Oslo, Oslo, Norway; 4 Institute of Health and Society, Department of Interdisciplinary Health Sciences, University of Oslo, Oslo, Norway

**Keywords:** Physical activity, Acculturation, Pregnancy, Postpartum

## Abstract

There are few studies of the migration context factors and physical activity (PA) level among minority ethnic women in Europe. We investigated the association between migration context factors and moderate to vigorous physical activity (MVPA) among minority ethnic women. Objectively recorded MVPA were obtained from 487 minority ethnic women included in the STORK-Groruddalen Cohort Study at three time points in pregnancy/postpartum. We investigated the associations between (a) contact with ethnic Norwegians and (b) Norwegian language skills and. No associations were observed in pregnancy. Postpartum, women who reported contact with ethnic Norwegians accumulated 17 MVPA min/day (95% CI: -.60, 34.54) more than women with no contact. In complete case analyses, this difference was significant (27 MVPA min/day (95% CI: 8.60, 44.54)). In early postpartum women with contact with ethnic Norwegians seems to be more physically active than women without contact. No associations were observed in pregnancy.

**Supplementary Information:**

The online version contains supplementary material available at 10.1007/s10903-023-01535-9.

## Background

Physical activity (PA) levels are reported to decrease during pregnancy and postpartum [1]. Western European women in Norway spend more time in moderate-to-vigorous physical activity (MVPA) in pregnancy than ethnic minority women, and by 3 months postpartum, their MVPA had increased significantly contrasted with women with ethnic South Asian background (182 MVPA minutes/week (bouts ≥ 10 MVPA min)) [2]. Increased PA level reduces the risk of adverse health outcomes (e.g., gestational diabetes, postpartum depression, cardio-vascular disease) [3, 4], which are more pronounced in ethnic minority groups [5–8]. Thus, it is important to understand factors that influence MVPA in ethnic minority women to inform health promotion.

Women’s PA behavior is influenced by a number of factors and their interactions [9] such as ethnicity [10, 11], previous PA level [1], socio-economic position [12], children [10], health status [13], PA friendliness of the neighborhood [2], cultural norms and low level of health literacy [14–16] and national/local political context [17, 18]. Further, the migration context, including acculturation, is an additional PA determinant for ethnic minorities [9]. Psychological acculturation has been defined as the result of intra-individual change processes resulting when person moving into a new cultural environment [19]. Acculturation may involve shifts in attitudes, behavior and social factors, thus, a growing body of research on PA in ethnic minority groups centers on acculturation [20].

Epidemiological studies often use simple acculturation proxies as it is not feasible to employ comprehensive acculturation instruments. According to Doucerain and co-workers [19] studies that employ simple proxies should avoid referring to these as measures of acculturation, but refer to specific domains of acculturation, also supported by Bornstein [21]. The current study is based on these recommendations.

Two commonly studied domains of acculturation are language and social contact with individuals with ethnic majority background [22]. Skills in the majority language may enhance ethnic minority women’s health literacy and ability to find, understand and use health information, and make informed health choices [23]. Social contact with individuals with ethnic majority background may positively influence PA, given that such contact provides health information and social support for PA [24, 25], and given that ethnic majority individuals have adopted a higher PA level (descriptive norms) [26, 27].

There are few studies of the association between acculturation and PA during pregnancy and postpartum. In studies of pregnant Latina women in US, a high level of acculturation (measured as preference of English language) was associated with meeting PA guidelines [28] and participation in sports/exercise [29]. In contrast, Latinas with a low level of acculturation (preference of Spanish language) reported more frequent household/caregiving PA [29]. Studies based on objective measures of MVPA from the postpartum period show that Latina women in the US who preferred Spanish language were more physically active [30]. In contrast, for Hispanic women in the US, no association was observed between an overall acculturation-score, based on media language preference (Spanish or English) and social contact with the majority population (Americans), and objectively recorded MVPA [31]. Cross-sectional studies of non-pregnant Latino/Hispanic US resident women of the association between acculturation and self-reported PA [32] and objectively recorded PA [30, 33–37] yielded inconsistent findings.

We are not aware of studies of the association between acculturation and PA in pregnancy or early postpartum in Europe, but there are cross-sectional studies in non-pregnant women showing a consistent positive association between various acculturation proxies and self-reported leisure-time PA [38, 39], but not with PA-intensity level [40, 41].

Our aims were to investigate if the two specific acculturation domains (language skills and social contact with members of majority group) were associated with PA level in ethnic minority women. Our research questions were [1] is social contact with the majority population or [2] Norwegian language skills associated with total MVPA min/day, during pregnancy and postpartum among ethnic minority women [3]? Are the associations modified by stage of pregnancy or early postpartum period? We hypothesized that [1] social contact with ethnic majority women and [2] level of Norwegian language skills were positively associated with MVPA during pregnancy/postpartum.

## Methods

### Study Design, Population/Setting and Data Collection

Data was obtained from the population-based STORK-G cohort study of pregnant women from Oslo, Norway. Pregnant women attending three child health clinics for antenatal care were recruited between May 2008 and May 2010. Trained midwives conducted face-to face interviews at the child health clinics at visit 1 (mean gestational week 15, early pregnancy), visit 2 (mean gestational week 28, late pregnancy), and visit 3 (mean 14 weeks postpartum). MVPA was objectively recorded immediately following each visit. Information material and questionnaires were translated to eight languages: Arabic, English, Sorani, Somali, Tamil, Turkish, Urdu, and Vietnamese, covering the largest ethnic groups in Oslo [42]. In addition, a professional translator assisted during interviews if required. Inclusion criteria were [1] living in one of the three collaborating city districts, [2] planned to give birth at collaborating hospitals, [3] in gestational week ≤ 20, [4] not suffering from diseases necessitating intensive hospital follow-up during pregnancy, [5] not already included with a pregnancy lasting ≥ 22 weeks, and [6] able to communicate in Norwegian or any of the other eight languages. Study methods are described in detail elsewhere [42]. Written informed consent was obtained from all participants ahead of the study. The Regional Committee for Medical and Health Research Ethics for South Eastern Norway (ref: 2007/894) and the Norwegian Data Inspectorate approved the study protocol [42]. Only study participants who were born abroad, or born in Norway with two immigrant parents, were eligible in the current study.

### Primary Outcome: Moderate to Vigorous Physical Activity

Total MVPA (min/day) was recorded by the SenseWear™ Pro3 Armband [43] (SWA) at visit 1–3 [44–47]. At each visit, women were asked to wear the SWA across the right triceps brachii 24 h per day over 4–7 days and remove it only for water activities. Raw data was integrated into 60-seconds epochs using the manufacturer’s software [48]. The summed value of 1-min epochs was used to estimate metabolic equivalents (METs). MVPA were defined as minute epochs ≥ 3METs. One valid SWA day was defined as ≥ 19.2 h/day of wear time, and SWA data from each visit was valid given ≥ 2 valid SWA days.

### Main Exposures

#### Contact with Ethnic Norwegians

Data on cross-cultural social contact was collected at visit 1 and operationalized by two items that measured frequency of visits by ethnic Norwegians during the last year (item 1) or frequency of help from ethnic Norwegians (item 2). The response alternatives were never, seldom, weekly, and daily. Women who reported “never” on both items were categorized as having no contact, while women who reported seldom, weekly or daily on at least one item were categorized as having contact with ethnic Norwegians.

#### Self-Reported Norwegian Language Skills

Self-reported Norwegian language skills at visit 1 was rated as poor, not very good, fair, good and very good. A binary variable reflecting low skill level (poor and not very good) and a high skill level (fair, good and very good) was used in the analyses.

### Confounders and Other Baseline Characteristics

Confounders were age, body mass index (BMI) kg/m^2^, education, and ethnicity (Supplementary Fig. 1). Age was treated as a continuous outcome. BMI was obtained from Tanita-BC 418 MA (Tanita, Tokyo, Japan). Highest educational level categories were primary school or less (≤ 10 years), high school (10–12 years) and university or college. Ethnicity was defined by the participant’s country of birth, or that of her mother if born outside Europe or North America [42]. Ethnic categories were South Asian origin, Middle Eastern origin and other.

## Statistical Methods

### Descriptive Analyses

Descriptive data are presented as mean, standard deviation (SD), or frequencies and proportions, for participants with and without valid PA data across time-points (Table 1).

**Table 1:** Characteristic of the cohort with and without valid physical activity (PA) data at three timepoints

		Visit 1^1^		Visit 2^2^		Visit 3^3^	
	Total	Valid PA	No PA data	Valid PA	No PA data	Valid PA	No PA data
	n = 487	n = 358	n = 129	n = 300	n = 187	n = 169	n = 318
Age (years) (mean/SD)	29.1 (5.0)	29.2 (4.8)	29.0 (5.4)	29.3 (5.1)	28.8 (4.8)	29.5 (4.9)	28.9 (5.0)
Body mass index kg/m^2^ (mean/SD)	25.4 (5.0)	25.0 (4.5)	26.4 (6.1)	25.2 (5.0)	25.7 (5.0)	25.3 (5.0)	25.4 (5.0)
Time of residence (years) (mean/SD) *	9.5 (7.9)	9.4 (8.1)	9.5 (7.2)	9.4 (7.8)	9.6 (8.0)	9.9 (8.3)	9.2 (7.7)
Marital status*, n (%)							
Married/cohabitant	456 (94)	335 (94)	121 (94)	285 (95)	171 (91)	161 (95)	295 (93)
Single	31 (6)	23 (6)	8 (6)	15 (5)	16 (9)	8 (5)	23 (7)
Ethnic origin, n (%)							
South Asia	200 (41)	153 (43)	47 (36)	125 (42)	75 (40)	69 (41)	131 (41)
Middle East	126 (26)	93 (26)	33 (26)	75 (25)	51 (27)	42 (25)	84 (27)
Other ethnicities	161 (33)	112 (31)	49 (38)	100 (33)	61 (33)	58 (34)	103 (32)
Education*, n (%)							
Primary school or less	123 (25)	91 (25)	32 (25)	77 (26)	46 (25)	43 (26)	80 (25)
High school/secondary	221 (46)	163 (46)	58 (46)	136 (46)	85 (46)	69 (41)	152 (48)
College/University	139 (29)	103 (29)	36 (29)	85 (28)	54 (29)	56 (33)	83 (27)
Occupation*, n (%)							
Don’t have any	170 (35)	125 (35)	45 (36)	104 (35)	66 (36)	54 (32)	116 (37)
Elementary occupations	62 (13)	45 (13)	17 (13)	38 (13)	24 (13)	22 (13)	40 (13)
Clerical service and assembly occupation	170 (35)	127 (35)	43 (34)	106 (35)	64 (34)	61 (36)	109 (34)
Managers and degree occupations	82 (17)	61 (17)	21 (17)	51 (17)	31 (17)	32 (19)	50 (16)
Parity, n (%)							
Nulliparous	205 (42)	155 (43)	50 (39)	124 (41)	81 (43)	68 (40)	137 (43)
Parous	285 (58)	203 (57)	79 (61)	176 (59)	106 (57)	101 (60)	181 (57)
Minority ethnic women, n (%)							
Born abroad	436 (90)	315 (88)	121 (94)	269 (90)	167 (89)	152 (90)	284 (89)
Born in Norway	51 (10)	43 (12)	8 (6)	31 (10)	20 (11)	17 (10)	34 (11)
Translator, n (%)							
yes	108 (22)	81 (23)	27 (21)	73 (24)	35 (19)	40 (24)	68 (21)
no	379 (78)	277 (77)	102 (79)	227 (76)	152 (81)	129 (76)	250 (79)

Values are n (%) unless otherwise stated

^1^ mean gestational week 15, ^2^ mean gestational week 28, ^3^ mean postpartum week 14

^a^ Valid PA data defined as ≥ 2 days with 19.2 h/day recorded with SenseWear Armband™ Pro3

*Missing: time of residence n = 39, marital status n = 39 visit 2 and n = 110 visit 3, education n = 4, occupation n = 3

### Analyses of the Associations

In separate statistical models we analyzed the associations between each main exposure and MVPA. We used mixed effects linear regression analyses with random intercepts. Level 1 data consisted of repeated measures of MVPA (visits 1–3), nested within women at level 2. Level 2 was treated as random effects in the analyses. In model 1, we employed the visit number (visit 1–3) to model time, but to control for variation in gestational and postpartum week of recording PA, we controlled for gestational/postpartum week centered at the mean week at each visit. We controlled for SWA wear-time mean-centered at each visit and included an interaction term between visit and main exposure to investigate time-varying association with MVPA. In model 2, we additionally adjusted for the confounders age, BMI, educational level, and ethnicity. To assess effect-modification, we explored separate demographic variables in two-way interaction terms with acculturation: (a) ethnicity, (b) parity (binary categories: nullipara and uni-/multipara), and (c) migration status (binary categories born abroad and born in Norway with two immigrant parents).

Few women had missing data on the two main exposures (2.1–5.3%). The sample percentage with missing PA data was 26.5%, 38.4% and 65.3% at visit 1, 2 and 3, respectively (Table 1). Reasons for missing PA data were not attending study visit, not accepting to wear SWA, or < 2 days wear time. Given the proportion of missing data, and to strengthen the plausibility of the missing-at-random assumption, we performed multiple imputation by chained equations [49]. We used predictive mean matching and accounted for dependencies between repeated measures of MVPA using the MICE package in RStudio version 1.1.419 [50]. We generated 50 imputed datasets with 100 burn-in iterations. We performed all other analyses in STATA 15, including multiple imputation analyses (MIA) using the STATA command mi estimate to obtain pooled estimates based on mixed effects regression analyses across the 50 imputed datasets [50]. We present estimates from MIA in the paper, while estimates from complete-case-analyses (CCA) are presented in text if diverting from the MIA (49).

In Supplementary Tables 1, complete CCA results are presented. Sensitivity analyses were performed to assess confounding effects of occupational status, previous PA level, self-reported health, and immigration status in separate models to avoid overadjustment. The results from sensitivity analyses were consistent with main results (data not shown).

## Results

### Sample Characteristics

**Fig. 1:**
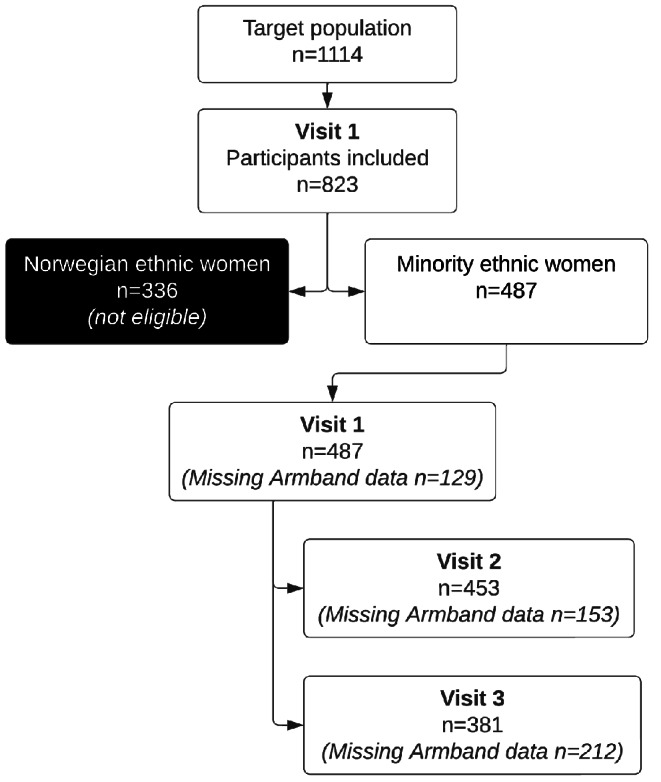
Flowchart of study sample and drop-out

The study sample consisted of 487 ethnic minority women included at visit 1 (Fig. 1).

The mean (SD) age was 29.1 (5.0) years and BMI was 25.4 (5.0) kg/m^2^. 90% were born outside Norway, and 22% needed a professional translator at study visits. The mean (SD) week for SWA monitoring was gestational week 15.6 (3.5) at visit 1 and gestational week 28.3 (1.5) at visit 2, and postpartum week 13.9 (2.5) at visit 3. The mean (SD) SWA wear-time ranged from 23.3 to 23.5 (0.5-0.7) hours across visits. There were minor differences between women with and without valid PA data at visit 1, but the latter had higher BMI and a larger proportion had ethnic background from South Asia (Table 1).

### Descriptive Analyses of MVPA During Pregnancy and Postpartum

Mean (SD) MVPA min/day was 78.0 (62.4) in early pregnancy, 63.6 (51.1) min/day in late pregnancy and 80.7 (63.9) min/day at postpartum. MVPA declined from early pregnancy to late pregnancy for women with low and high levels of contact with ethnic Norwegians and Norwegian language skills. MVPA increased postpartum across sub-groups, except for women without contact with ethnic Norwegians, where the mean value did not change (Table 2).

**Table 2:** Descriptive analyses of moderate to vigorous physical activity min/day by levels of main exposures

Main exposures		Visit 1^1^		Visit 2^2^		Visit 3^3^	
	Total		MVPA^a^ min/day		MVPA^a^ min/day		MVPA^a^ min/day
	n (%)	n (%)	Mean (SD)	n (%)	Mean (SD)	n (%)	Mean (SD)
Contact with ethnic Norwegians*							
No contact	108 (23)	82 (24)	73.9 (57.4)	77 (27)	63.4 (61.5)	39 (24)	62.9 (46.2)
Contact	353 (77)	260 (76)	77.8 (59.6)	208 (73)	63.0 (48.0)	123 (76)	85.3 (68.6)
Norwegian language skills**							
Low	99 (21)	69 (19)	82.3 (64.8)	63 (21)	65.3 (67.0)	34 (20)	74.7 (61.0)
Medium/high	378 (79)	286 (81)	76.8 (62.3)	234 (79)	63.1 (47.6)	132 (80)	81.3 (64.9)

^1^ mean gestational week 15, ^2^ mean gestational week 28, ^3^ mean Postpartum week 14

^a^ MVPA = moderate to vigorous physical activity

*Missing: n = 26

** Missing: n = 10

### Associations Between Specific Domains of Acculturation and MVPA

In pregnancy, there were small and non-significant differences in MVPA between women with and without contact with ethnic Norwegians and Norwegian language skills (Table 3, Model 2). Estimates obtained from CCA and MIA agreed. In postpartum, there were non-significant MVPA differences between women with low versus high Norwegian language skills in MIA and CCA. Women with social contact with ethnic Norwegians accumulated an additional 17 MVPA min/day (95% CI: − 0.60, 34.54, p = 0.058) in MIA (Table 3). The difference was borderline significant in MIA, and statistically significant in CCA, which indicated women with social contact with ethnic Norwegians accumulated an additional 27 MVPA min/day (95% CI: 8.60, 44.54, p = 0.004) (Supplementary Table 1) compared with women without contact. Parity, ethnicity, and migration status did not modify the associations between the two acculturation domains and MVPA in the MIA (results not presented).

**Table 3:** Association between main exposures and moderate-to-vigorous physical activity. (Mixed effect linear regression analyses of imputed data)

			Group difference in MVPA^a^ min/day					
			Visit 1^1^		Visit 2^2^		Visit 3^3^	
Main exposure	Model	***n***	β	(95% CI)	β	(95% CI)	β	(95% CI)
Contact with ethnic Norwegians (ref. no contact)								
Contact	M1	487	2.4	(-11.88, 16.62)	3.1	(-11.16, 17.37)	14.9	(-2.78, 32.58)
	M2		4.4	(-9.28, 18.15)	5.2	(-8.73, 19.11)	17.0	(-0.60, 34.54)
Norwegian language skills (ref. low)								
medium/high	M1	487	-2.7	(-17.03, 11.73)	-3.7	(-19.36, 12.04)	0.3	(-19.01, 19.57)
	M2		0.0	(-13.76, 13.77)	-1.0	(-16.25, 14.17)	2.9	(-15.90, 21.77)

^1^ mean gestational week 15, ^2^ mean gestational week 28, ^3^ mean postpartum week 14

M1 = adjusted time, SenseWear armband monitoring week and weartime

M2 = additionally adjusted for age, body mass index, educational level and ethnicity

^a^ MVPA = Moderate to vigorous physical activity recorded by SenseWear Armband Pro3

## Discussion

To the best of our knowledge, this is the first study of associations between specific domains of acculturation and objectively recorded MVPA in pregnancy and postpartum in a cohort of ethnic minority women in Europe. Contact with ethnic Norwegians and Norwegian language skills were not associated with MVPA in pregnancy. At postpartum, there is some support for claiming that women with contact with ethnic Norwegians are more physically active than those with no contact.

### Associations in Pregnancy

Previous studies have indicated an association between preference for the majority language and self-reported PA in pregnancy among immigrants [28, 29, 51]. A plausible reason that the current study indicates no difference in *total* MVPA in pregnancy is that negative and positive associations with different types of PA (e.g., household and recreational domains) demonstrated elsewhere [29], are “evened out”. Positive associations between acculturation and *self-reported* exercise demonstrated in previous studies [28, 51] are prone to social desirability bias, and may explain why the current analyses of objectively recorded MVPA did not replicate previous findings. The conflicting findings may also result from analyses of different domains of acculturation, different national contexts, and different migrant populations [52–54].

The absence of associations between social contact/language skills and MVPA during pregnancy, imply that pregnancy-specific barriers common among ethnic minority women such as cultural norms (e.g., discouraging physical activity in pregnancy, obligation to perform domestic task), safety concern for the fetus (e.g., lack of information), and health issues (e.g., nausea, muscle pain, anthropometric changes) [14, 55], reduce the impact of other factors determining PA levels.

### Associations in Early Postpartum

The CCA and MIA indicate that ethnic minority women having cross-cultural accumulate 27 and 17 MVPA min/day more than women with no cross-cultural contact, although borderline significant in MIA. Postpartum MVPA duration obtained objectively, was notably greater in Mexian-American women with limited cross-cultural social contact in childhood compared to their highly exposed counterparts [30]. The different operationalization of cross-cultural contact in the two studies may partly explain the conflicting results. Another reason may be that the Norwegian context differs from the US context; the *maternity leave period* and the *social arena* created by the public maternity care services by offering pram walk groups in Norway may promote PA and explain the difference with respect to US-based studies [30] Another study of Hispanic women in the US indicated no association between acculturation and MVPA [31]. However, acculturation was measured using a *multidimensional acculturation scale* with acculturation expressed as a single score [31], which may camouflage different associations between separate acculturation domains and MVPA [19] investigated in the current study.

The positive association observed postpartum between cross-cultural social contact and MVPA may be mediated through different mechanisms: As Western women are generally more active [2], social contact with Western women may give access to health information concerning PA, while descriptive norms and social support for doing PA may also promote PA in ethnic minority women [26, 27]. A possible explanation for the positive influence of cross-cultural contact on MVPA in postpartum may be that the mechanisms of social contact are more pronounced at postpartum since a clear shift in PA engagement among Western women occur at this timepoint [2]. Further, it may also suggest sensitivity to situation-specific demands (e.g., cultural norms) revealing dynamic adaptability within specific domains [14, 21]: In the postpartum period, cultural norms for PA may be more aligned between the new culture and the inherited culture, leading to a greater psychological impact of cross-cultural contact and increased PA levels, than during pregnancy [14].

Although, our study revealed that cross-cultural contact may influence postpartum PA, promotion of PA among ethnic minority women in pregnancy and postpartum must be informed by evidence of multiple PA determinants and their interaction sensitive to ethnic groups [10, 14, 56–61]. Future intervention studies are warranted to provide stronger evidence of the magnitude of the effect of promoting cross-cultural contact in antenatal health care services.

### Strengths and Limitations

This study has several strengths. The models are based on objectively recorded MVPA at multiple time points in pregnancy and postpartum, thus, MVPA estimates are not prone to recall [62] or social desirability bias [63]. Use of interpreters and translated study material promoted inclusion of women with poor Norwegian language skills and strengthened the sample’s representativeness [9, 64], and enhanced the study’s potential to investigate if associations differ by ethnic groups and immigration status. Analyses of specific acculturation domains acknowledge that different domains may influence PA differently [19, 21].

The study has some limitations. The specific measures of acculturation domains are not validated. The study does not capture how individuals balance heritage and host culture within the domain [21, 22]. Another limitation is the large proportion of participants with missing PA data. To mitigate potential bias, we performed multiple imputation, and, for transparency purposes, we have presented the CCA in supplementary material [49, 65]. Small group sizes in some subgroups reduces the statistical power. We included women with two valid SWA days, which is below the recommended cut-off (≥ 3 days) [63]. This may have negatively influenced the reliability of the MVPA estimates [65]. Additionally, wearing an activity monitor may have influenced the participants’ PA behavior.

## Conclusion

Cross-cultural contact or skills in the majority population’s language were not associated with MVPA during pregnancy. However, the results indicated that having cross-cultural contact may have a positive influence on MVPA in early postpartum.

### Electronic supplementary material

Supplementary Material 1

Supplementary Material 2

## Data Availability

Due to ethical restrictions and patient confidentiality, not all data can be made publicly available. Data are available upon request from the Medical Faculty at the University of Oslo for researchers who meet the criteria for access to confidential data. Access can be arranged by direct request to co-author Anne Karen Jenum (a.k.jenum@medisin.uio.no).

## Abbreviations

MVPA: Moderate-to-vigorous physical activity
PA: Physical activity
SD: Standard deviation
95% Cl: 95% confidence interval
SWA: SenseWear™ Armband
CCA: Complete case analyses
MIA: Multiple imputation analyses

## References

[1] Condello G, Puggina A, Aleksovska K, Buck C, Burns C, Cardon G (2017). Behavioral determinants of physical activity across the life course: a “DEterminants of DIet and physical ACtivity” (DEDIPAC) umbrella systematic literature review. Int J Behav Nutr Phys Act.

[2] Richardsen KR, Mdala I, Berntsen S, Ommundsen Y, Martinsen EW, Sletner L (2016). Objectively recorded physical activity in pregnancy and postpartum in a multi-ethnic cohort: association with access to recreational areas in the neighbourhood. Int J Behav Nutr Phys Act.

[3] Nakamura A, van der Waerden J, Melchior M, Bolze C, El-Khoury F, Pryor L (2019). Physical activity during pregnancy and postpartum depression: systematic review and meta-analysis. J Affect Disord.

[4] Dipietro L, Evenson KR, Bloodgood B, Sprow K, Troiano RP, Piercy KL (2019). Benefits of physical activity during pregnancy and Postpartum: an Umbrella Review. Med Sci Sports Exerc.

[5] Neupane D, McLachlan CS, Sharma R, Gyawali B, Khanal V, Mishra SR (2014). Prevalence of hypertension in member countries of south asian association for Regional Cooperation (SAARC): systematic review and meta-analysis. Med (Baltim).

[6] Jenum AK, Mørkrid K, Sletner L, Vange S, Torper JL, Nakstad B (2012). Impact of ethnicity on gestational diabetes identified with the WHO and the modified International Association of diabetes and pregnancy study groups criteria: a population-based cohort study. Eur J Endocrinol.

[7] Shakeel N, Sletner L, Falk RS, Slinning K, Martinsen EW, Jenum AK (2018). Prevalence of postpartum depressive symptoms in a multiethnic population and the role of ethnicity and integration. J Affect Disord.

[8] Shakeel N, Eberhard-Gran M, Sletner L, Slinning K, Martinsen EW, Holme I (2015). A prospective cohort study of depression in pregnancy, prevalence and risk factors in a multi-ethnic population. BMC Pregnancy Childbirth.

[9] Langøien LJ, Terragni L, Rugseth G, Nicolaou M, Holdsworth M, Stronks K (2017). Systematic mapping review of the factors influencing physical activity and sedentary behaviour in ethnic minority groups in Europe: a DEDIPAC study. Int J Behav Nutr Phys Act.

[10] Babakus WS, Thompson JL (2012). Physical activity among south asian women: a systematic, mixed-methods review. Int J Behav Nutr Phys Act.

[11] Crespo CJ, Smit E, Andersen RE, Carter-Pokras O, Ainsworth BE (2000). Race/ethnicity, social class and their relation to physical inactivity during leisure time: results from the Third National Health and Nutrition Examination Survey, 1988–1994. Am J Prev Med.

[12] De Munter JSL, Agyemang C, Brewster LM, Stronks K, Van Valkengoed IGM (2012). The association of leisure-time physical activity and active commuting with measures of socioeconomic position in a multiethnic population living in the Netherlands: results from the cross-sectional SUNSET study. BMC Public Health.

[13] Bauman AEP, Reis RSP, Sallis JFP, Wells JCP, Loos RJFP, Martin BWMD (2012). Correlates of physical activity: why are some people physically active and others not?. Lancet.

[14] Angrish K, Khan-Poulin Y, Mangat J, Mack DE, Nagpal TS. Culturally tailored strategies for prenatal physical activity for south asian women: a scoping review. J Immigr Minor Health. 2023. 10.1007/s10903-023-01486-1 37193874

[15] Gele AA, Pettersen KS, Torheim LE, Kumar B (2016). Health literacy: the missing link in improving the health of somali immigrant women in Oslo. BMC Public Health.

[16] Le C, Pettersen HS, joranger KS, P., Guttersrud Ø. Health literacy in five immigrant populations in Norway: Pakistan, Somalia, Turkey, and Vietnam. English Summary. *In* Helsekompetansen i fem innvandrerpopulasjoner i Norge: Pakistan, Polen, Somalia, Tyrkia og Vietnam. Befolkningens helsekompetanse, del II. The Norwegian Directorate of Health; 2021.

[17] Benn T, Pfister G (2013). Meeting needs of muslim girls in school sport: case studies exploring cultural and religious diversity. Eur J Sport Sci.

[18] Dagkas S, Benn T (2006). Young Muslim women’s experiences of Islam and physical education in Greece and Britain: a comparative study. Sport Educ Soc.

[19] Doucerain MM, Segalowitz N, Ryder AG. Acculturation measurement: from simple proxies to sophisticated toolkit. In: Schwartz SJ, Unger JB, editors. The Oxford Handbook of Acculturation and Health. Oxford University Press; 2017. pp. 97–117.

[20] Abraido-Lanza AF, Flórez KR. Acculturation and Physical Activity among Latinos. In: Schwartz SJ, Unger JB, editors. The Oxford Handbook of Acculturation and Health. Oxford University Press; 2017. pp. 343–55.

[21] Bornstein MH (2017). The specificity Principle in Acculturation Science. Perspect Psychol Sci.

[22] Arends-Tóth J, van de Vijver FJR, Sam DL, Berry JW, Sam DL, Berry JW (2006). Assessment of psychological acculturation. The Cambridge Handbook of Acculturation psychology. Cambridge Handbooks in psychology.

[23] Buja A, Rabensteiner A, Sperotto M, Grotto G, Bertoncello C, Cocchio S (2020). Health literacy and physical activity: a systematic review. J Phys Act Health.

[24] Holt-Lunstad J, Uchino B. Social support and Health. In: Glanz K, Rimer, B.K., and Viswanath, K., editor. Health Behavior Theory, Research, and Practice. 5th ed2015. p. 183-5.

[25] Thomas W, Karen. Glanz BKR, Viswanath K (2015). Valente. Social Network and health behaviors. Health Behavior.

[26] Rivis A, Sheeran P (2003). Descriptive norms as an additional predictor in the theory of planned behaviour: a meta-analysis. Curr Psychol.

[27] Fishbein MA (2010). I. Predicting and changing behavior.

[28] Gollenberg A, Pekow P, Markenson G, Tucker KL, Chasan-Taber L (2008). Dietary behaviors, physical activity, and cigarette smoking among pregnant puerto rican women. Am J Clin Nutr.

[29] Chasan-Taber L, Schmidt MD, Pekow P, Sternfeld B, Manson J, Markenson G (2007). Correlates of physical activity in pregnancy among Latina women. Matern Child Health J.

[30] Joseph RP, Benitez TJ, Ainsworth BE, Todd M, Keller C (2018). Acculturation and Physical Activity among Latinas enrolled in a 12-Month walking intervention. West J Nurs Res.

[31] Martin CL, Tate DF, Schaffner A, Brannen A, Hatley KE, Diamond M (2017). Acculturation Influences Postpartum Eating, Activity, and Weight Retention in Low-Income Hispanic Women. J Womens Health (Larchmt).

[32] Benitez TJ, Dodgson JE, Coe K, Keller C (2016). Utility of Acculturation in Physical Activity Research in Latina adults: an integrative review of literature. Health Educ Behav.

[33] Camplain R, Sotres-Alvarez D, Alvarez C, Wilson R, Perreira KM, Castañeda SF (2020). The association of acculturation with accelerometer-assessed and self-reported physical activity and sedentary behavior: the Hispanic Community Health Study/Study of Latinos. Prev Med Rep.

[34] Salinas JJ, Hilfinger Messias DK, Morales-Campos D, Parra-Medina D (2014). English language proficiency and physical activity among mexican-origin women in South Texas and South Carolina. J Health Care Poor Underserved.

[35] Zan H, Fan JX (2018). Reporting more but moving less? The Complex relationship between Acculturation and Physical Activity among US adults. Am J Health Promot.

[36] Perez LG, Chavez A, Marquez DX, Soto SC, Haughton J, Arredondo EM (2017). Associations of Acculturation with Self-Report and Objective Physical Activity and sedentary behaviors among Latinas. Health Educ Behav.

[37] Marquez DX, McAuley E (2006). Gender and acculturation influences on physical activity in latino adults. Annals of behavioral medicine: a publication of the Society of Behavioral Medicine.

[38] Hosper K, Klazinga NS, Stronks K (2007). Acculturation does not necessarily lead to increased physical activity during leisure time: a cross-sectional study among turkish young people in the Netherlands. BMC Public Health.

[39] Jonsson LS, Palmer K, Ohlsson H, Sundquist J, Sundquist K (2012). Is acculturation associated with physical activity among female immigrants in Sweden?. J Public Health (Oxf).

[40] Koca C, Lapa TY (2014). Analysis of physical activity and acculturation among turkish Migrants in Germany and England. Percept Mot Skills.

[41] Hosper K, Nierkens V, van Valkengoed I, Stronks K (2008). Motivational factors mediating the association between acculturation and participation in sport among young turkish and moroccan women in the Netherlands. Prev Med.

[42] Jenum AK, Sletner L, Voldner N, Vangen S, Morkrid K, Andersen LF (2010). The STORK Groruddalen research programme: a population-based cohort study of gestational diabetes, physical activity, and obesity in pregnancy in a multiethnic population. Rationale, methods, study population, and participation rates. Scand J Public Health.

[43] Body M, Inc. Pittsburg, PA, USA.

[44] Berntsen S, Hageberg R, Aandstad A, Mowinckel P, Anderssen SA, Carlsen KH (2010). Validity of physical activity monitors in adults participating in free-living activities. Br J Sports Med.

[45] Berntsen S, Stafne SN, MØRkved SIV (2011). Physical activity monitor for recording energy expenditure in pregnancy. Acta Obstet Gynecol Scand.

[46] Johannsen DL, Andres Calabro M, Stewart J, Franke W, Rood JC, Welk GJ (2010). Accuracy of Armband Monitors for Measuring Daily Energy expenditure in healthy adults. Med Sci Sports Exerc.

[47] Brazeau A-S, Beaudoin N, Bélisle V, Messier V, Karelis AD, Rabasa-Lhoret R (2014). Validation and reliability of two activity monitors for energy expenditure assessment. J Sci Med Sport.

[48] SenseWear™. Professional Research Software. Version 6.1. BodyMedia Inc.

[49] Sterne JAC, White IR, Carlin JB, Spratt M, Royston P, Kenward MG (2009). Multiple imputation for missing data in epidemiological and clinical research: potential and pitfalls. BMJ.

[50] White IR, Royston P, Wood AM (2011). Multiple imputation using chained equations: issues and guidance for practice. Stat Med.

[51] Wilkie G, Leung K, Moore Simas TA, Tucker KL, Chasan-Taber L. The Association between Acculturation and Diet and physical activity among pregnant hispanic women with abnormal glucose tolerance. Journal of Women’s Health; 2022. 10.1089/jwh.2022.0017 PMC9805839 36040352

[52] Schwartz SJ, Unger JB, Zamboanga BL, Szapocznik J (2010). Rethinking the Concept of Acculturation: implications for theory and research. Am Psychol.

[53] Fox M, Thayer Z, Wadhwa PD (2017). Assessment of acculturation in minority health research. Soc Sci Med.

[54] Fox M, Thayer ZM, Wadhwa PD (2017). Acculturation and Health: the moderating role of Sociocultural Context: Context Moderates how Acculturation affects Health. Am Anthropol.

[55] Harrison AL, Taylor NF, Shields N, Frawley HC (2018). Attitudes, barriers and enablers to physical activity in pregnant women: a systematic review. J Physiother.

[56] Makama M, Awoke MA, Skouteris H, Moran LJ, Lim S (2021). Barriers and facilitators to a healthy lifestyle in postpartum women: a systematic review of qualitative and quantitative studies in postpartum women and healthcare providers. Obes Rev.

[57] Ryan RA, Lappen H, Bihuniak JD (2022). Barriers and facilitators to healthy eating and physical activity Postpartum: a qualitative systematic review. J Acad Nutr Diet.

[58] Thompson EL, Vamos CA, Daley EM (2017). Physical activity during pregnancy and the role of theory in promoting positive behavior change: a systematic review. J Sport Health Sci.

[59] Garland M, Wilbur J, Semanik P, Fogg L (2019). Correlates of physical activity during pregnancy: a systematic review with implications for evidence-based practice. Worldviews Evid Based Nurs.

[60] Eyler AA, Matson-Koffman D, Rohm Young D, Wilcox S, Wilbur J, Thompson JL (2003). Quantitative study of correlates of physical activity in women from diverse racial/ethnic groups: women’s Cardiovascular Health Network project–introduction and methodology. Am J Prev Med.

[61] O’Driscoll T, Banting LK, Borkoles E, Eime R, Polman R (2014). A systematic literature review of sport and physical activity participation in culturally and linguistically diverse (CALD) migrant populations. J Immigr Minor Health.

[62] Guerin E, Ferraro ZM, Adamo KB, Prud’homme D. The need to objectively measure physical activity during pregnancy: considerations for Clinical Research and Public Health Impact. Matern Child Health J; 2018. 10.1007/s10995-018-2475-4 29411253

[63] Dowd KP, Szeklicki R, Minetto MA, Murphy MH, Polito A, Ghigo E et al. A systematic literature review of reviews on techniques for physical activity measurement in adults: a DEDIPAC study. Int J Behav Nutr Phys Act. 2018;15(1). 10.1186/s12966-017-0636-2 PMC5806271 29422051

[64] Bull FC, Al-Ansari SS, Biddle S, Borodulin K, Buman MP, Cardon G (2020). World Health Organization 2020 guidelines on physical activity and sedentary behaviour. Br J Sports Med.

[65] Stephens SP, Beyene JP, Tremblay MSP, Faulkner GP, Pullnayegum EP, Feldman BMMDMF (2018). Strategies for dealing with Missing Accelerometer Data. Rheum Dis Clin North Am.

