# Supplementary material for: Cross-Cultural Contact and Norwegian Language Skills Among Ethnic Minority Women in Norway, and Relationship with Physical Activity in Pregnancy and Postpartum: The STORK-Groruddalen Cohort Study
Source: J Immigr Minor Health. 2023 Aug 28;26(1):63–71. doi: 10.1007/s10903-023-01535-9 (PMC10771598; doi:10.1007/s10903-023-01535-9)
Supplement: Supplementary file 1 — Supplementary Material 1 [file 10903_2023_1535_MOESM1_ESM.docx]

| Supplementary Table 1. Association between main exposure and moderate-to-vigorous physical activity.  (Mixed effect linear regression analyses of complete cases) | | | | | | | | | | | |
| --- | --- | --- | --- | --- | --- | --- | --- | --- | --- | --- | --- |
|  |  |  |  | Group difference in MVPA (mean min/day)^a^ | | | | | | | |
|  |  |  |  | Visit 1^1^ | |  | Visit 2^2^ | |  | Visit 3^3^ | |
| Main exposure |  | Model | ***n*** | β | (95% CI) |  | β | (95% CI) |  | β | (95% CI) |
| Contact with ethnic Norwegians  (ref. no) |  |  |  |  |  |  |  |  |  |  |  |
| social contact |  | M1 | 342 | 3.1 | (-10.82, 16.93) |  | -.1 | (-14.56, 14.34) |  | **25.6** | **(6.92, 44.34)** |
|  |  | M2 |  | 5.7 | (-7.38, 18.74) |  | 2.7 | (-10.90, 16.33) |  | **26.6** | **(8.60, 44.54)** |
| Norwegian language skills (ref. low) |  |  |  |  |  |  |  |  |  |  |  |
| medium/high |  | M1 | 354 | -3.7 | (-18.85, 11.45) |  | -1.8 | (-17.53, 13.99) |  | 7.2 | (-12.97, 27.43) |
|  |  | M2 |  | -1.1 | (-15.51, 13.33) |  | -1.8 | (-16.91, 13.32) |  | 6.5 | (-13.25, 26.30) |

^1^ mean gestational week 15, ^2^ mean gestational week 28, ^3^ mean postpartum week 14

M1 = Model 1 adjusted for SenseWear armband week and weartime

M2 = Model 2 additionally adjusted for age, body mass index, educational level and ethnicity

^a^ MVPA = Moderate to vigorous physical activity recorded by SenseWear Armband Pro3

Missing: Contact with ethnic Norwegians n=26 and self-reported language skills n=10

Bold text indicate statistical significance
