# Supplementary figures and images for: Cross-Cultural Contact and Norwegian Language Skills Among Ethnic Minority Women in Norway, and Relationship with Physical Activity in Pregnancy and Postpartum: The STORK-Groruddalen Cohort Study
Source: J Immigr Minor Health. 2023 Aug 28;26(1):63–71. doi: 10.1007/s10903-023-01535-9 (PMC10771598; doi:10.1007/s10903-023-01535-9)

# Supplementary Figure 1: Directed Acyclic Graph (DAG)


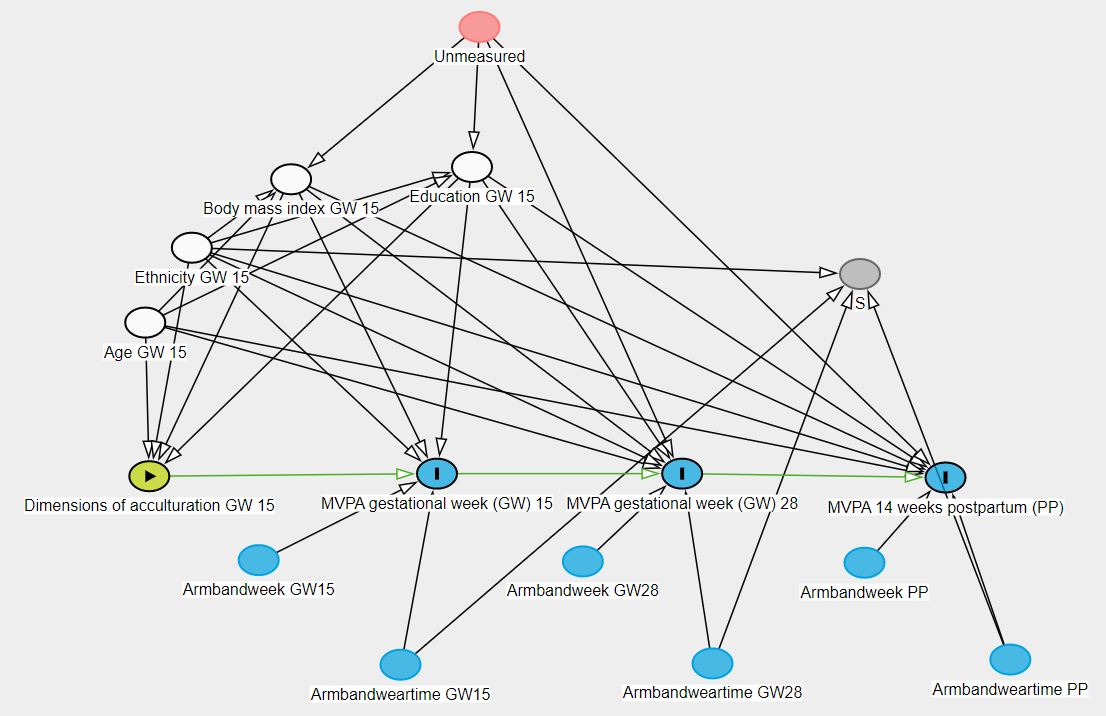


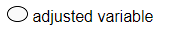

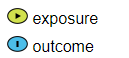


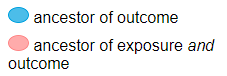


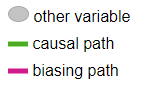

Supplement: Supplementary file 2 — Supplementary Material 2 [file 10903_2023_1535_MOESM2_ESM.docx]
